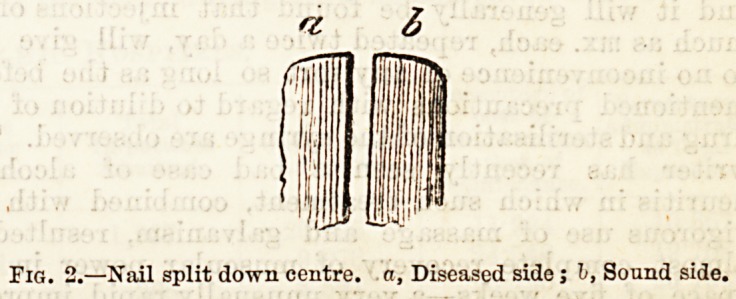# Ingrowing Toe-Nail

**Published:** 1893-09-30

**Authors:** G. Munro Smith

**Affiliations:** Senior Assistant Surgeon, Royal Infirmary, Bristol


### INGROWING TOE-NAIL.

By G. Munro Smith, M.R.C.S., L.R.C.P.Lond., Senior Assistant Surgeon, Royal Infirmary, Bristol.

I.—Pathology.

Although this is one of the most distressing and painful of minor surgical ailments, it cannot be said that its pathology has been satisfactorily explained. Early in the present century a great deal was written about it, but of late years the text-books have contained very little on the subject, and much of this seems incorrect. No doubt its name has been responsible for erroneous ideas as to its nature, for in most cases it is neither "ingrowing" nor "incurvated." Treves describes it as a hypertrophy of the nail, which grows laterally, and causes injurious pressure on the skin. Ashurst and Mansell Moullin only devote half-a-dozen lines to its pathology. Agnew is a little fuller; he says, "The causes of incurvated toe-nail are threefold—namely, narrow and tightly-fitting shoes, paring the corners of the nails too closely, and the accumulation of desquamated epidermis under the edges." Godlee says much the same in Holmes's and Hulke's "System of Surgery." In an article on this affection in the "Bristol Medico-Chirurgical Journal" for June, 1884, Mr. Greig Smith points out that many causes may produce the disease, such as careless cutting, redundance of flesh in the toes, pressure of badly-fitting boots, flattening of the arch of the foot, eversion of the great toe, and inversion of the lesser toes.

In all the above descriptions, it seems, however, that one point is omitted—namely, the necrosis of the nail on the affected side. It will be found that the adhesions between the nail and its groove or "bed" on one side are absent, and a probe can be easily inserted between the dead portion and the surrounding granulations, which sprout up round it as they would round any lifeless structure. This edge of nail, however, is not only lifeless, but is sharp and rough, and is well calculated to cause the greatest pain by its constant irritation of the sensitive tissues into which it presses.

To make this clear, it will be necessary to say a word or two on the structure of the parts concerned. The nail, as is well known, is composed originally of the *stratum lucidum*, the true horny layer of the skin covering it in early embryonic life, and persisting in most people as the thin fold of epidermis partly covering the "lunula." The deeper portion consists of the malpighian layer, and this fits closely on to the matrix or papillary layer of the epidermis. Laterally the cells which compose the nail are directly continuous with the surrounding epidermis, and are bathed with nourishing fluid which permeates between them and keeps them alive. The actual connection can be seen in the diagram (Fig. 1), modified from a drawing by Ranvier, where (*a*) represents the superficial cells, (*b*)

Fig. 1.—*f*, Loose epidermic scales; *g*, Thickened epidermis. The darkened line (*e*) represents the stratum granulunum which is absent in the nail itself.

the deeper malpighian layers, (*c*) the connecting link between the nail and the overhanging skin at the side, and (*d*) the vascular matrix. It will be noted that the epidermis adjacent to the edge is considerably thickened, and might exert considerable pressure if squeezed against the nail.

There is therefore a vital connection between the lateral margin of the nail and the skin. Any undue pressure, whether from a boot or any other cause, might easily sever this band of living tissue. The same pressure continued would cause the underlying blood-vessels in the papillæ of the dermis to be so squeezed as to starve the part. The result would be the death of this portion of the nail. The condition called "in-growing toe-nail" follows this almost as a matter of course. The necrosed portion loses its lustre and smoothness, and becomes white or yellow and opaque. Its sharp, rough edge irritates the neighbouring matrix, the papillæ of which spring up and enlarge, as granulations. The nail, in fact, acts as a foreign body. In milder cases the accumulation of dead epidermic scales appear to act injuriously in the same manner as the pressure of the boot, interfering with proper nutrition.

If the above be a correct view of the pathology of the disease, what ought one to find in the removal of the offending cause? Firstly, there should certainly be no hypertrophy. As a matter of fact small nails are the most apt to be associated with this condition; and examination of the two halves will show that they are really of the same size in almost every case. Real incurvation does sometimes occur, but rarely. The diseased half comes away easily, the sound half requires much more tearing. The drawing (Fig. 2) shows the difference, the unshaded portion (the diseased side) being rough and yellowish white, very different from the sound part.

The true nature of this painful affection would therefore appear to be a *localised necrosis* of part of the nail, caused by the severance of delicate vascular and vital connections by undue pressure. This pres-

Fig. 2.—Nail split down centre. *a*, Diseased side; *b*, Sound side.

sure being caused by tight boots, malformation of the toes, unequal growth of the nail itself, and in rare cases real incurvation; never, probably, a primary "ingrowing."

## Figures and Tables

**Fig. 1. f1:**
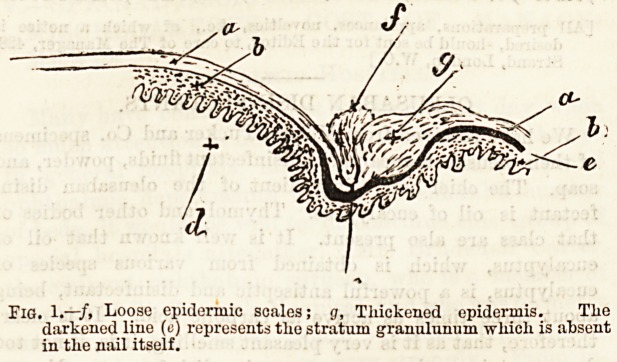


**Fig. 2. f2:**